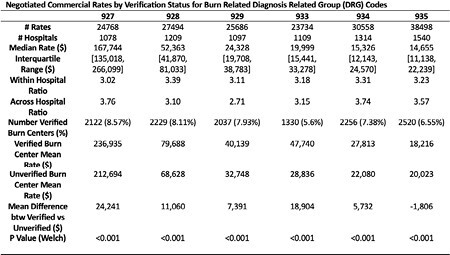# 100 Negotiated Commercial Rates for Burn Care in the Era of Price Transparency—Does Verification Signal Quality?

**DOI:** 10.1093/jbcr/irae036.099

**Published:** 2024-04-17

**Authors:** Eloise Stanton, Akshay Swaminathan, Nada Rizk, Rachel Pedreira, Clifford C Sheckter

**Affiliations:** Keck School of Medicine of USC, LOS ANGELES, CA; Stanford University, Stanford, California; Harvard University, Boston, Massachusetts; Stanford University, Menlo Park, California; Stanford/Santa Clara Valley Medical Center, San Jose, CA; Keck School of Medicine of USC, LOS ANGELES, CA; Stanford University, Stanford, California; Harvard University, Boston, Massachusetts; Stanford University, Menlo Park, California; Stanford/Santa Clara Valley Medical Center, San Jose, CA; Keck School of Medicine of USC, LOS ANGELES, CA; Stanford University, Stanford, California; Harvard University, Boston, Massachusetts; Stanford University, Menlo Park, California; Stanford/Santa Clara Valley Medical Center, San Jose, CA; Keck School of Medicine of USC, LOS ANGELES, CA; Stanford University, Stanford, California; Harvard University, Boston, Massachusetts; Stanford University, Menlo Park, California; Stanford/Santa Clara Valley Medical Center, San Jose, CA; Keck School of Medicine of USC, LOS ANGELES, CA; Stanford University, Stanford, California; Harvard University, Boston, Massachusetts; Stanford University, Menlo Park, California; Stanford/Santa Clara Valley Medical Center, San Jose, CA

## Abstract

**Introduction:**

The Price Transparency Rule was enacted in 2021 to facilitate disclosure of negotiated rates between payors and hospitals with the goal of reducing the cost of healthcare. Burn care is intensive and costly; to date there have been no national analyses of price variation. In particular, we aimed to investigate the relationship between prices, market competition and American Burn Association (ABA) verification status.

**Methods:**

All available commercial rates for 2021-2022 for Diagnosis Related Group (DRG) codes 927 (extensive burn with grafting, mechanical ventilation >96 hours), 928 (extensive burn with grafting or inhalation injury with complication/comorbidity), 929 (extensive burn with grafting or inhalation injury without complication/comorbidity), 933 (extensive burn without grafting, mechanical ventilation >96 hours), 934 (extensive burn without grafting, no mechanical ventilation), and 935 (non-extensive burn without grafting) were merged with hospital level variables, burn center verification status, and Herfindahl-Hirschman Index (HHI, marker of healthcare market competition). For each DRG code, a linear random effects model was fit with cost as the outcome and the following variables as covariates: HHI (categorical), plan type (categorical), safety net status (binary), nonprofit status (binary), verified burn center status (binary), rural status (binary), teaching hospital status (binary). A random effect was included for each unique facility to respect endogenous variation with hospitals.

**Results:**

There were 170,738 rates published from 1541 unique hospitals. Rates for the same DRG varied by a factor of 3 within hospitals. Similarly, rates across hospitals varied by a factor of 3 for all DRGs, with DRG 927 having the most variation. ABA verification status was independently associated with higher reimbursement rates adjusting for facility level factors for all DRGs except for 935. Notably HHI was the greatest predictor of commercial rates, suggesting that market competition is the dominant force in determining burn center payments.

**Conclusions:**

Regional market competition was the dominant factor in determining burn care remuneration. ABA verification status for burn centers was independently associated with increased payments, suggesting a signal for leverage/quality.

**Applicability of Research to Practice:**

For the first time we have national benchmarks for inpatient burn commercial rates which vary substantially within and between hospitals. Burn centers may leverage this information when negotiating rates. In particular, verification status garners greater payments.